# Transient Dexmedetomidine Bolus-Induced Excessive Urination Intraoperatively in a 68-Year-Old Male

**DOI:** 10.1155/2020/6660611

**Published:** 2020-12-02

**Authors:** Joseph A. McGuire, Summer A. Niazi, Daniel C. Sizemore

**Affiliations:** ^1^West Virginia University School of Medicine, Morgantown, WV, USA; ^2^West Virginia University Department of Anesthesiology, Medical Center Drive, Morgantown, WV 26505, USA

## Abstract

Excessive urination can be a perioperative challenge for providers due to the possibility of secondary hypernatremia. Dexmedetomidine has previously been reported by several groups to induce a polyuric-like syndrome; however, the exact mechanism in humans remains unclear. In this report, we discuss a case of intraoperative, transient dexmedetomidine bolus-induced excessive urination and suggest a potential mechanism by which this may occur in a subset of the population.

## 1. Introduction

Dexmedetomidine (Precedex) is a strong agonist of α2-adrenergic receptors that induces sedation without causing respiratory depression. It achieves this by increasing the activity of gamma-aminobutyric acid (GABA) neurons in the ventrolateral preoptic nucleus of the brain [1]. Side effects of this medication include hypotension, bradycardia, or dry mouth [2]; however, it is reported by several groups that dexmedetomidine may also induce polyuric syndrome in some patients [1,3–6]. It is believed that this occurs through the inhibition of arginine-vasopressin (AVP) release [6]. Written consent, in accordance with the Health Insurance Portability and Accountability Act (HIPPA), was obtained.

## 2. Case Presentation

A 68-year-old male patient presented for the removal of an intramedullary nail (IMN) from his right femur and possible total knee arthroplasty (TKA) for his dislocated right knee following a traumatic event. His past medical history is significant for hypertension, hyperlipidemia, hyperkalemia, benign prostatic hyperplasia with urinary hesitancy, atrial fibrillation, a crush injury that caused a closed fracture of multiple ribs on the right side and the pelvis, and tobacco use. The patient's surgical history includes a colonoscopy and multiple orthopedic surgeries. His previous surgeries did not have any history of abnormal urine output or sodium levels. The patient denied complaints of frequent urination; however, his history included a complaint of urinary hesitancy. His medications included aspirin, atorvastatin, celecoxib, and oxycodone, and a medication allergy to losartan. Preoperative laboratory tests indicated slightly low sodium levels and slightly elevated glucose levels, but a well-functioning renal system. On physical exam, height was 1.753 m and weight 116.9 kg (body mass index of 38.14).

**Figure 1:**
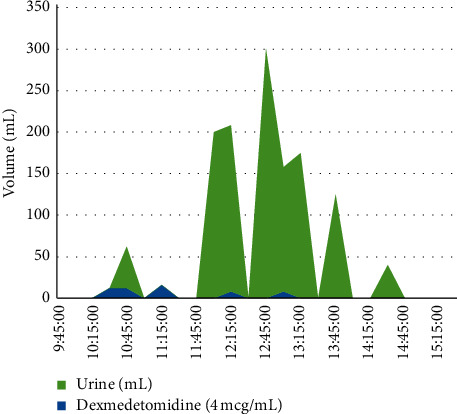
Dexmedetomidine effect on urine output over time. The correlation between dexmedetomidine boli and urine output is shown. Volumes of both urine and dexmedetomidine are shown. Dexmedetomidine volumes are also listed in the text.

On the day of surgery, the anesthesia team planned for spinal block (hyperbaric bupivacaine, 2 mL of 0.75%) and monitored anesthesia care (MAC) with a propofol infusion. The patient was given 2 mg of midazolam preoperatively. Supplemental oxygenation was provided and standard American Society of Anesthesiologists (ASA) monitors were utilized for continuous monitoring. Total volume of 1500 mL of Lactated Ringer's (LR) premix infusion was given intraoperatively. The surgery lasted a total of 5 hours and 32 minutes and the patients urine output for the duration of the operation totaled 1240 mL, 975 mL of which were excreted within a two-hour span and hour and a half following the delivery of the boli of dexmedetomidine (12 mcg, 12 mcg, and 16 mcg) (Figure 1).

The postoperative period was uncomplicated, the patient maintained normal sodium levels and normal electrolytes despite frequent monitoring in the immediate period after surgery.

## 3. Discussion

Polyuria is defined as the excretion of 40 mL/kg/24 h [7]. During the case, we reviewed the drugs given and discussed if they had evidence of inducing polyuria. In addition to dexmedetomidine, the patient received propofol, ondansetron, ephedrine, phenylephrine, cefazolin, tranexamic acid, bupivacaine, and LR premix infusion intraoperatively, none of which have evidence in the literature of having a diuretic effect. In fact, propofol [8], ondansetron, ephedrine [9], phenylephrine [10], and bupivacaine [8] have all been shown to induce urinary retention. The patient had no history of renal disease, diabetes insipidus, or thyroid disease and he was on no medication which would have a diuretic effect. He had slightly elevated potassium levels intraoperatively, but otherwise, all of his electrolytes were normal. In his previous surgery, almost all of the same anesthetic drugs were used, other than dexmedetomidine, ephedrine, phenylephrine, tranexamic acid, and bupivacaine; yet, excessive urination was not noted. Spinal cord trauma has been shown to lead to excessive urine output [11]; however, there were no clinical signs indicating a spinal cord injury whatsoever.

Dexmedetomidine was administered at the following volumes and corresponding times: 12 mL, 10:30; 12 mL, 10:45; 16 mL, 11:15; 8 mL, 12:15; 8 mL, 13:00. The last spike in urinary output was recorded at 14:30. The half-life of dexmedetomidine has been reported as approximately two hours [12]. All of the instances of excessive urination were recorded within the previously reported half-life times, indicating a greater efficacy at higher concentrations of the drug.

In a literature review, a report by Shirasaka et al. seems to provide a mechanism of action of the diuretic effect of dexmedetomidine. The authors showed in rat models that dexmedetomidine dose-dependency inhibited the magnocellular cells of the paraventricular nucleus, which are known to secrete vasopressin and oxytocin [13]. They explain that it achieves this through activation of the G protein-coupled receptor leading to an inwardly rectifying potassium current. The inhibition of vasopressin would lead to diuresis, considering the potent antidiuretic effects it normally has in the body.

Most cases reporting dexmedetomidine-induced polyuria have occurred in patients undergoing posterior spinal fusion surgery [14]. While there is no clear answer, increased potassium levels, made available by surgical iatrogenic myolysis, could provide an explanation for the drug's ability to induce polyuria.

## 4. Conclusions

While it is not an effect seen in the entirety of the population, here we report that dexmedetomidine may cause excessive urination in certain individuals. Considering the possibility of hypernatremia secondary to excessive urination, clinicians should be aware of this. Further studies to find the exact mechanism of action need to be done in order to determine if certain individuals are more susceptible than others through a genetic mechanism. Dexmedetomidine use may help prevent anesthetic complication of urinary retention and further study is warranted.

## Data Availability

The data used to support the findings of this study are included within the article.
